# Association between Dietary Patterns and Kidney Function Parameters in Adults with Metabolic Syndrome: A Cross-Sectional Study

**DOI:** 10.3390/nu13010040

**Published:** 2020-12-24

**Authors:** Ahmad Syauqy, Chien-Yeh Hsu, Hsiu-An Lee, Hsiao-Hsien Rau, Jane C.-J. Chao

**Affiliations:** 1 School of Nutrition and Health Sciences, College of Nutrition, Taipei Medical University, 250 Wu-Hsing Street, Taipei 11031, Taiwan; syauqy@fk.undip.ac.id; 2 Department of Nutrition Science, Faculty of Medicine, Diponegoro University, Jl. Prof. H. Soedarto, SH., Tembalang, Semarang 50275, Indonesia; 3 Department of Information Management, National Taipei University of Nursing and Health Sciences, 365 Ming-Te Road, Peitou District, Taipei 11219, Taiwan; cyhsu@ntunhs.edu.tw; 4 Master Program in Global Health and Development, College of Public Health, Taipei Medical University, 250 Wu-Hsing Street, Taipei 11031, Taiwan; 5 Department of Computer Science and Information Engineering, Tamkang University, 151 Yingzhuan Road, Tamsui District, New Taipei City 25137, Taiwan; billy72325@gmail.com; 6 Joint Commission of Taiwan, 5F, 31, Section 2, Sanmin Road, Banqiao District, New Taipei City 22069, Taiwan; hh.rau@jct.org.tw; 7 Nutrition Research Center, Taipei Medical University Hospital, 252 Wu-Hsing Street, Taipei 11031, Taiwan

**Keywords:** dietary patterns, kidney function, metabolic syndrome, cross-sectional study, Taiwan

## Abstract

This study explored the association between dietary patterns and kidney function parameters in adults with metabolic syndrome in Taiwan. This cross-sectional study was undertaken in 56,476 adults from the health screening centers in Taiwan from 2001 to 2010. Dietary intake and dietary patterns were assessed using a food frequency questionnaire and principal component analysis, respectively. Blood urea nitrogen (BUN), creatinine, estimated glomerular filtration rate (eGFR), and uric acid were measured as clinical parameters of kidney function. Multivariate linear regression was conducted to explore the relationship between dietary patterns and kidney function parameters. After adjusting for confounders, the highest tertiles of the processed food–sweets dietary pattern and the meat–seafood–eggs dietary pattern were associated with increased BUN, creatinine, and uric acid but decreased eGFR (all adjusted *p* < 0.05). Meanwhile, the highest tertiles of the veggie–fruit–grains dietary pattern and the milk–dairy dietary pattern were associated with decreased BUN, creatinine, and uric acid but increased eGFR (all adjusted *p* < 0.05). A processed food–sweets dietary pattern or a meat–seafood–eggs dietary pattern is associated with worse kidney function parameters in adults with metabolic syndrome. In contrast, a veggie–fruit–grains dietary pattern or a milk–dairy dietary pattern is associated with better kidney function parameters.

## 1. Introduction

Metabolic syndrome is defined as a cluster of metabolic disorders such as central obesity, increased fasting blood glucose (FBG), elevated blood pressure (BP), reduced high-density lipoprotein (HDL) cholesterol, and increased triglycerides [1]. Metabolic syndrome and its components have been linked to many diseases, especially chronic kidney disease (CKD) [2,3,4]. A meta-analysis study reported that metabolic syndrome was correlated with the development of CKD [5]. Individuals with metabolic syndrome had higher odds of having CKD progression than those who had normal metabolic conditions [5]. Moreover, the prevalence of metabolic syndrome has been significantly increased in Taiwan [6] and many countries [7,8]. Simultaneously, the incidence of CKD was also considerably raised in Taiwan [9] along with increased metabolic syndrome [10]. Hence, it is important to determine the risk of impaired kidney function in a population with metabolic syndrome to decrease the prevalence or incidence of CKD.

Epidemiological studies demonstrated that diet had a significant role in the rapidly growing prevalence of metabolic syndrome [11,12,13] and CKD [14,15,16,17]. When assessing the relationship between diet and disease, a dietary pattern containing complex food may be more accurate than a single nutrient or food item, as it presents a broader picture of dietary intake [11]. The Western diet considered as an unhealthy dietary pattern was positively correlated with metabolic syndrome [11,12] and CKD [14,15,16,17]. A cohort study reported that high intakes of meat and instant food characterized by a diet rich in saturated fat, sodium, and sugar were correlated with a rapid decline of estimated glomerular filtration rate (eGFR) [18]. The prudent diet and the traditional diet characterized by plant-based foods, grains, fruit, vegetables, fresh foods, and boiled/steamed foods thought to be healthy dietary patterns were inversely associated with metabolic syndrome [11,12,13] and CKD [15,16,17]. The main components of these healthy dietary patterns are high fiber foods and a no-oil cooking method. Middle-aged or elderly adults at high cardiovascular disease risk who consumed a Mediterranean diet were associated with a reduction of cardiometabolic risk factors and the improvement of metabolic syndrome [19,20]. Moreover, patients with diabetic kidney disease consuming a diet replacing saturated fat, trans fat, or cholesterol with monounsaturated or polyunsaturated fat were correlated with more favorable kidney function outcomes [21].

A study has investigated the relationship between diet and kidney dysfunction in a healthy population [18]. A Western dietary pattern was correlated with increased odds of a rapid decline in kidney function, while a Dietary Approaches to Stop Hypertension (DASH)-style dietary pattern protected against rapid kidney function decline [18]. A previous meta-analysis study described that a reduction in protein consumption could delay CKD progression in type 2 diabetic patients [22]. A healthy diet rich in fish and vegetables may improve kidney function among the diabetic population in Taiwan [23]. However, to the best of our knowledge, very few studies have previously investigated the association between dietary patterns and kidney function in a population with metabolic syndrome. Therefore, the aim of this study was to examine the association between dietary patterns and kidney function parameters in adults with metabolic syndrome in Taiwan.

## 2. Materials and Methods

### 2.1. Subjects and Study Design

We used the cross-sectional data collected by the Mei Jau (MJ) health screening centers from 2001 to 2010. The MJ Group, a private institution in Taiwan, collected data including demographic data, lifestyle, dietary data, and biochemical data from people who came to their health screening centers for a regular health check-up. The subjects agreed to fill in the informed consent form and authorized the MJ to use the data for study purposes. Details of the MJ data collection procedures have been reported elsewhere [24]. In this study, a total of 87,831 individuals aged ≥20 years met the criteria of metabolic syndrome from the MJ database between 2001 and 2010. Then, we excluded 20,270 subjects who were pregnant or had cancer, liver disease, lung disease, dialysis, or eGFR <15 mL/min/1.73 m^2^ [25]. Furthermore, the subjects who had missing data (*n* = 11,085) were also excluded. Finally, a total of 56,476 subjects were analyzed for the study. The study was approved by Joint Institutional Review Board of Taipei Medical University (N201802065).

### 2.2. Definition of Metabolic Syndrome

We used the International Diabetes Federation Task Force on Epidemiology and Prevention (a Joint Interim Statement in 2009) to define metabolic syndrome. Individuals who had three or more of the following conditions: waist circumference ≥ 90 cm in men or ≥80 cm in female (Asian/Taiwan population), triglycerides ≥ 1.7 mmol/L (or using drug treatment for blood triglycerides abnormality), HDL cholesterol < 1.0 mmol/L in men or <1.3 mmol/L in women (or using drug treatment for HDL cholesterol abnormality), systolic BP ≥ 130 mmHg or diastolic BP ≥ 85 mmHg (or using antihypertensive drug treatment or the patient with a history of hypertension), and FBG ≥ 5.6 mmol/L (or using drug treatment for blood glucose abnormality) [1].

### 2.3. Assessment of Dietary Intake

Dietary intake was evaluated by self-administered food frequency questionnaire (FFQ). The questionnaire is a validated tool as well described in the previous studies [11,26]. The FFQ had 22 food groups representing the characteristics of dietary intake in Taiwan. The FFQ provided frequency and portion size of food intake with pictures, as described previously [11]. One example question regarding the consumption of eggs was, “How many servings of eggs do you eat? (One serving is equal to one chicken egg, one duck egg, or five quail eggs.)”, and the answers had five options including none or <1 serving/week, 1–3 servings/week, 4–6 servings/week, 1 serving/day, and 2 servings/day. The scores of food intake were defined as 1 to 5 from the lowest to the highest frequency [11,26]. Food items and description of servings in each food group are shown in Table S1.

### 2.4. Assessment of Clinical Parameters and Biochemical Data

The measurements of anthropometric data and blood pressure as well as the collection of fasting blood samples were performed by the trained nurses in the MJ health screening centers. Weight and height were assessed using an anthropometer. Body mass index (BMI) was evaluated as weight (kg) divided by the square of height (m^2^). Waist circumference was assessed at the midpoint between the top of the hip bone and the bottom of the ribs. Blood pressure was measured on the right arm by a sphygmomanometer (Citizen CH-5000, Tokyo, Japan). Plasma HDL cholesterol, low-density lipoprotein (LDL) cholesterol, triglycerides, FBG, blood urea nitrogen (BUN), creatinine, uric acid, and C-reactive protein (CRP) levels were analyzed enzymatically using an auto-analyzer (Hitachi 7150, Tokyo, Japan) [27]. The value of eGFR was computed using creatinine, age, sex, and race as the parameters based on the equation of the Chronic Kidney Disease Epidemiology Collaboration [28].

### 2.5. Assessment of Other Variables

Information on demographic data and lifestyle including age, sex, marital status, level of education, alcohol and smoking status, and level of physical activity were collected using a self-administered questionnaire [25]. Sex was dichotomized as men and women. Marital status was defined as never married, married, and divorce. Level of education was simply categorized as low (below senior high school, equivalent to <9 school years) and high (senior high school and above, equivalent to ≥9 school years). Current smoking and drinking status were dichotomized as yes and no. Physical activity was defined as low (<2 h/week) and high (2 h/week).

### 2.6. Statistical Analysis

We used principal component analysis (PCA) with eigenvalues (>1) in orthogonal rotation to derive dietary patterns [29]. Factor loadings of ≥|±0.3| were considered to classify dietary patterns [30,31]. The factor scores of dietary patterns for each subject were computed by summing up the intake of each food group weighted by its factor loading, and then we classified the scores into tertiles. The dietary pattern was named by which ones had higher factor loadings. To analyze the differences in demographic and lifestyle variables across tertiles of dietary patterns, we used the chi-square test for categorical variables and one-way analysis of variance with Bonferroni post-hoc test for continuous variables. The analysis of covariance was performed to examine the relationship between dietary patterns and kidney function parameters, and we counted the mean value (95% CI) of kidney function parameters for the subjects with different dietary patterns after adjusting for demographic data, lifestyle, and components of metabolic syndrome. Multivariate linear regression was conducted to explore the relationship between dietary patterns and kidney function parameters after adjusting for multiple confounding variables in two models. Model 1 was adjusted for demographic data and lifestyle (sex, age, marital status, level of education, drinking, smoking, and level of physical activity). In model 2, we included the components of metabolic syndrome (waist circumference, systolic and diastolic BP, HDL cholesterol, triglycerides, and FBG). The value of *p* < 0.05 was considered statistically significant. All statistical analyses were performed using SPSS 24 (IBM Corp., Armonk, NY, USA).

## 3. Results

### 3.1. Characteristics of the Subjects

Table 1 demonstrates the characteristics of all the subjects. Among 56,476 subjects with metabolic syndrome, 31.3% were female, 24.0% were smokers, 16.7% were drinkers, and 32.9% had high physical activity. 

### 3.2. Dietary Patterns

Table 2 describes four dietary patterns derived by PCA. The four dietary patterns were identified as the processed food–sweets dietary pattern, veggie–fruit–grains dietary pattern, meat–seafood–eggs dietary pattern, and milk–dairy dietary pattern with 55.67% of the total variance (36.53%, 8.73%, 5.34%, and 5.07%, respectively). The processed food–sweets dietary pattern contained eight food groups: processed food, deep-fried food, sugary drinks, sauce, instant noodles, jam/honey, refined dessert, and fried rice/flour products. The veggie–fruit–grains dietary pattern included eight food groups: dark- or light-colored vegetables, fruit, root crops, vegetables in oil/dressing, whole grains, rice/flour products, and legumes/soy products. The meat–seafood–eggs dietary pattern contained four food groups: meat, seafood, organ meats, and eggs. The milk–dairy dietary pattern included two food groups: milk and dairy products.

### 3.3. Characteristics of the Subjects across Tertiles of Dietary Patterns

Table 3, Table 4, Table 5 and Table 6 show the characteristics of the subjects across tertiles of each dietary pattern. In comparison with the subjects in the lowest tertile (T1) of the processed food–sweets dietary pattern, the subjects in the highest tertile (T3) tended to be younger, have higher proportions of females, married, smokers, and drinkers, have lower proportions of high education level and high physical activity, and have worse metabolic parameters and inflammation (all *p* < 0.05) (Table 3). Moreover, the subjects in the highest tertile of the processed food–sweets dietary pattern were associated with higher BUN, creatinine, and uric acid levels but lower eGFR (all *p* < 0.05) compared with those in the lowest tertile.

The subjects in the highest tertile of the veggie–fruit–grains dietary pattern tended to be older, have higher proportions of females, married, high education level, non-smokers, non-drinkers, and high physical activity, and have better metabolic parameters and inflammation compared with those in the lowest tertile (all *p* < 0.05) (Table 4). Additionally, the subjects in the highest tertile of the veggie–fruit–grains dietary pattern were associated with lower BUN, creatinine, and uric acid levels but higher eGFR (all *p* < 0.05) compared with those in the lowest tertile.

The subjects in the highest tertile of the meat–seafood–eggs dietary pattern was likely to be younger, have higher proportions of females, married, smokers, and drinkers, have lower proportions of high education level and high physical activity, and have worse metabolic parameters and inflammation (all *p* < 0.05) (Table 5). Moreover, the subjects in the highest tertile of the meat–seafood–eggs dietary pattern were associated with higher BUN, creatinine, and uric acid levels but lower eGFR (all *p* < 0.05) compared with those in the lowest tertile.

The subjects in the highest tertile of the milk–dairy dietary pattern was likely to be older, have higher proportions of females, married, high education level, smokers, drinkers, and high physical activity, and have better metabolic parameters and inflammation compared with those in the lowest tertile (all *p* < 0.05) (Table 6). Furthermore, the subjects in the highest tertile of the milk–dairy dietary pattern were associated with lower BUN, creatinine, and uric acid levels but higher eGFR (all *p* < 0.05) compared with those in the lowest tertile.

### 3.4. Association between Dietary Patterns and Kidney Function Parameters

Table 7 explores the multivariable-adjusted means of BUN, creatinine, eGFR, and uric acid as parameters of kidney function across tertiles of dietary patterns. Higher consumption of the processed food–sweets dietary pattern or the meat–seafood–eggs dietary pattern was positively associated with BUN, creatinine, and uric acid but negatively correlated with eGFR after adjusting for model 1 (sex, age, marital status, education, current smoking, current drinking, and physical activity) and model 2 (model 1 and the components of metabolic syndrome) (all adjusted *p* < 0.05). While higher consumption of the veggie–fruit–grains dietary pattern or the milk–dairy dietary pattern was negatively associated with BUN, creatinine, and uric acid but positively correlated with eGFR after adjusting for model 1 and model 2 (all adjusted *p* < 0.05).

## 4. Discussion

Our study showed that higher consumption of the processed food–sweets dietary pattern or the meat–seafood–eggs dietary pattern was associated with higher BUN, creatinine, and uric acid levels but lower eGFR. In contrast, higher consumption of the veggie–fruit–grains dietary pattern or the milk–dairy dietary pattern was correlated with decreased BUN, creatinine, and uric acid as well as increased eGFR. The average values of kidney function parameters were normal in this study, because we included the individuals with metabolic syndrome rather than those with impaired kidney function and only excluded those with eGFR < 15 mL/min/1.73 m^2^. The characteristics of the processed food–sweets dietary pattern were high in saturated fat, sugar, sodium, and monosodium glutamate, which were similar to an unhealthy diet [32]. Individuals who consumed more processed food and sweetened beverages with high fat and sugar may affect CKD progression. This dietary pattern was strongly correlated with increased mortality of CKD patients [16]. The Western diet high in processed food, red meat, and refined food or an unhealthy diet high in saturated fat was significantly associated with a rapid decline of eGFR (3 mL/min/1.73 m^2^ per year) [18] and the risk of hyperalbuminuria [33]. Moreover, a higher sodium intake over time was significantly correlated with elevated serum uric acid as a marker for impaired kidney function [34].

Similar to the processed food–sweets dietary pattern, the meat–seafood–eggs dietary pattern characterized by high protein and saturated fat was also related to an unhealthy diet. High intakes of meat, fish, eggs, and other non-dairy sources of protein might expedite kidney dysfunction among individuals with mild kidney insufficiency [35]. Subsequently, an animal study showed that a low protein diet (5% of protein) for 12 weeks prevented endogenous uric acid synthesis and ameliorated kidney tubular damage in streptozotocin-induced diabetic rats compared with a normal protein diet (18% of protein) [36]. Moreover, restricted protein intake to 0.7 g/kg daily was beneficial for the management of diabetic nephropathy [37]. A very low protein or low protein diet improved kidney function in patients with diabetic nephropathy and chronic kidney failure [38]. In contrast, a high protein diet was correlated with increased serum urea and urinary calcium excretion in overweight/obese or type 2 diabetic subjects compared with a normal or low protein diet [39].

In contrast, the veggie–fruit–grains dietary pattern with high intakes of dietary fiber, vitamins, minerals, antioxidants, and unsaturated fat in our study was similar to the prudent dietary pattern in another study [26]. High intakes of fruit, vegetables, and other plant-based food were correlated with lower mortality of CKD [16]. The vegetables and fish dietary pattern was associated with lower creatinine levels and increased eGFR values in type 2 diabetic patients [23]. These data suggested that antioxidants, dietary fiber, and phytochemicals in the vegetable-rich dietary pattern could play important roles in the management of kidney disease [23]. Furthermore, similar to the veggie–fruit–grains dietary pattern in this study, the Dietary Approaches to Stop Hypertension (DASH) diet was correlated with decreased creatinine levels and increased eGFR levels. The results indicated that the DASH diet, characterized by high intakes of vegetables, fruit, and whole grains but low intakes of sodium, sweetened beverages, and meat, could protect against declined kidney function [40]. The DASH diet also reduced serum uric acid in hyperuricemic subjects [41].

Comparable to the veggie–fruit–grains dietary pattern, the milk–dairy dietary pattern composed of milk and dairy products was inversely correlated with kidney damage. Although milk and dairy products were partially linked to the Western dietary pattern [42,43], the healthy or prudent dietary pattern such as breakfast or cereal–dairy dietary pattern also included milk and dairy products [11,44]. Calcium and vitamin D in milk or dairy products may contribute to the beneficial effects on kidney function and metabolic syndrome [45,46,47]. Similar to our findings, individuals who consumed milk one time daily had lower serum uric acid levels compared with those who did not drink milk, indicating that dairy consumption was negatively correlated with serum uric acid levels [48]. Moreover, some studies showed that dairy products had protective effects on the incidence of hypertension, diabetes, and metabolic syndrome, which were related to kidney dysfunction [49,50,51]. Intakes of energy and protein were related to the impairment of kidney function in the metabolic syndrome population [52]. However, exact energy or protein intake could not be accurately calculated from the FFQ in our study, and further investigations may be needed.

Furthermore, our results found small changes in kidney function parameters but statistical significance among the tertiles of the dietary pattern, suggesting that a different degree of adherence to a dietary pattern could be significantly associated with changes in kidney function parameters. After further adjusting for the covariates, our results showed that the means of kidney function parameters tended to change slightly; however, the association between dietary patterns and kidney function parameters was still significant. This finding indicated that dietary patterns were independently correlated with kidney function parameters among adults with metabolic syndrome. The total variance of four dietary patterns in this study was 55.67%, which only explained the variation of each dietary pattern and made no assumption about causality [53]. However, more studies are needed to examine the effects of diets on the development or progression of kidney disease in a population with metabolic syndrome.

This study has several strengths. To the best of our knowledge, this is the first study to discuss the association between dietary patterns and kidney function parameters in Taiwanese adults with metabolic syndrome. Second, we used a large sample size, which represented a population of interest. Third, this study applied dietary patterns determined by a posteriori method that reflected actual dietary habits. However, this study also has some limitations. First, the use of a posteriori-derived dietary patterns is the potential lack of reproducibility of study findings. The method of PCA involves the subjective determination of the number of factors to retain in factor analysis and the method of rotation, which results in population-specific dietary patterns with likely limited comparability. Second, although the questionnaire in our study had been validated among the Taiwanese population, there might be self-reporting bias associated with the self-administered questionnaire. Furthermore, we were unable to obtain dietary intake information precisely regarding how much specific nutrients were consumed using an FFQ, especially the intake of energy, protein, fat, and other nutrients, including sodium, which could be essential to control the potential confounders. It is important to take into account that energy intake was not considered while using PCA; therefore, adherence to a dietary pattern could increase with energy intake due to the wider variety of foods consumed by those with higher caloric intake. Moreover, albumin excretion ratio (AER) was not available in the database. A previous study has demonstrated that both eGFR and AER are the main parameters used in the clinical practice to assess kidney function [54]. These two markers provided complementary data for the definition of impaired kidney function, and only one of these two markers could not provide reliable information about kidney function [54]. We also did not have albuminuria available for analysis. Recently, both eGFR values and the presence of albuminuria can be used for the diagnosis of CKD [55]. We used current smoking and drinking status as yes or no and did not describe the duration of smoking or the level of drinking across tertiles of each dietary pattern. Finally, the cross-sectional nature of the study implicated that we could not determine the cause and effect of this association.

## 5. Conclusions

Our study shows that the dietary pattern has an association with kidney function in Taiwanese adults with metabolic syndrome. A processed food–sweets dietary pattern or a meat–seafood–eggs dietary pattern is correlated with higher BUN, creatinine, and uric acid levels but lower eGFR in adults with metabolic syndrome. On the contrary, a veggie–fruit–grains dietary pattern or a milk–dairy dietary pattern is associated with lower BUN, creatinine, and uric acid levels but higher eGFR. The longitudinal studies may be considered to further investigate the effects of dietary patterns on the progression of declined kidney function among individuals with metabolic syndrome.

## Figures and Tables

**Table 1:** Characteristics of all the subjects (*n* = 56,476) ^1^.

Characteristics	All Subjects
Age (years)	41.2 ± 11.8
Sex (% female)	31.3
Marital status (% married)	73.6
Education (% high)	83.4
Current smoker (% yes)	24.0
Current drinker (% yes)	16.7
Physical activity (% high)	32.9
Body mass index (kg/m^2^)	25.2 ± 3.5
Waist circumference (cm)	84.8 ± 11.8
Systolic blood pressure (mmHg)	122 ± 23
Diastolic blood pressure (mmHg)	77 ± 15
Total cholesterol (mmol/L)	5.18 ± 1.07
HDL cholesterol (mmol/L)	1.07 ± 0.64
LDL cholesterol (mmol/L)	2.99 ± 1.49
Triglycerides (mmol/L)	2.04 ± 1.16
FBG (mmol/L)	5.64 ± 1.46
BUN (mmol/L)	4.95 ± 1.40
Creatinine (μmol/L)	89.2 ± 27.2
eGFR (mL/min/1.73 m^2^)	85.2 ± 24.1
Uric acid (mmol/L)	0.38 ± 0.10
CRP (mg/dL)	0.22 ± 0.38

HDL: high-density lipoprotein; LDL: low-density lipoprotein; FBG: fasting blood glucose; BUN: blood urea nitrogen; eGFR: estimated glomerular filtration rate; CRP: C-reactive protein. ^1^ Values are presented as mean ± SD for continuous variables or % for categorical variables.

**Table 2:** Factor loadings of four dietary patterns identified by principal component analysis.

	Processed Food–Sweets Dietary Pattern	Veggie–Fruit–Grains Dietary Pattern	Meat–Seafood–Eggs Dietary Pattern	Milk–Dairy Dietary Pattern
Milk	−0.004	0.185	0.184	0.776
Dairy products	0.185	0.181	0.260	0.707
Eggs	0.297	0.179	0.605	0.260
Meat	0.323	0.118	0.753	0.125
Seafood	0.106	0.325	0.666	0.214
Organ meats	0.326	0.149	0.610	0.108
Legumes/soy products	0.284	0.441	0.396	0.193
Light-colored vegetables	0.089	0.807	0.251	0.069
Dark-colored vegetables	0.066	0.825	0.241	0.081
Vegetables in oil/dressing	0.217	0.595	0.404	−0.078
Fruit	0.098	0.640	0.117	0.308
Whole grains	0.238	0.576	−0.096	0.222
Root crops	0.332	0.599	0.092	0.236
Rice/flour products	0.336	0.473	0.400	0.308
Fried rice/flour products	0.534	0.349	0.160	0.060
Refined dessert	0.568	0.232	0.079	0.416
Jam/honey	0.640	0.218	−0.007	0.416
Sugary drinks	0.678	0.003	0.237	0.283
Deep-fried food	0.688	0.125	0.400	0.099
Processed food	0.696	0.171	0.259	−0.009
Instant noodles	0.658	0.183	0.159	0.048
Sauce	0.679	0.117	0.256	0.041

**Table 3:** Characteristics of the subjects across tertiles of the processed food–sweets dietary pattern (*n* = 56,476) ^1^.

	Processed Food–Sweets Dietary Pattern			
	T1 (*n* = 18,825)	T2 (*n* = 18,826)	T3 (*n* = 18,825)	*p*-Value
Age (years)	44.6 ± 13.3 ^a^	41.4 ± 11.8 ^b^	37.8 ± 10.5 ^c^	<0.001
Sex (% female)	25.7	29.7	38.1	<0.001
Marital status (% married)	64.6	71.8	77.1	<0.001
Education (% high)	89.5	84.5	66.3	<0.001
Current smoker (% yes)	14.9	23.4	31.2	<0.001
Current drinker (% yes)	13.1	17.1	19.4	<0.001
Physical activity (% high)	33.8	33.2	31.5	<0.001
Body mass index (kg/m^2^)	24.9 ± 3.7 ^a^	25.4 ± 3.6 ^a^	25.4 ± 3.7 ^a^	0.009
Waist circumference (cm)	83.6 ± 12.6 ^a^	84.9 ± 11.0 ^b^	85.3 ± 11.0 ^c^	<0.001
Systolic blood pressure (mmHg)	121 ± 22 ^a^	122 ± 23 ^b^	124 ± 24 ^c^	<0.001
Diastolic blood pressure (mmHg)	76 ± 14 ^a^	77 ± 15 ^b^	77 ± 15 ^c^	<0.001
Total cholesterol (mmol/L)	5.11 ± 1.07 ^a^	5.18 ± 1.08 ^b^	5.23 ± 1.15 ^c^	0.004
HDL cholesterol (mmol/L)	1.09 ± 0.62 ^a^	1.08 ± 0.67 ^a^	1.04 ± 0.63 ^b^	<0.001
LDL cholesterol (mmol/L)	2.90 ± 1.52 ^a^	2.95 ± 1.52 ^b^	3.04 ± 1.46 ^c^	<0.001
Triglycerides (mmol/L)	1.98 ± 1.10 ^a^	2.04 ± 1.12 ^b^	2.10 ± 1.41 ^c^	<0.001
FBG (mmol/L)	5.57 ± 1.26 ^a^	5.63 ± 1.74 ^b^	5.67 ± 1.41 ^b^	<0.001
BUN (mmol/L)	4.81 ± 1.31 ^a^	4.95 ± 1.39 ^b^	4.98 ± 1.78 ^c^	<0.001
Creatinine (mol/L)	86.6 ± 32.2 ^a^	89.6 ± 30.0 ^a^	90.5 ± 26.0 ^b^	<0.001
eGFR (mL/min/1.73 m^2^)	85.6 ± 21.0 ^a^	85.1 ± 22.2 ^b^	84.9 ± 22.6 ^b^	0.011
Uric acid (mmol/L)	0.36 ± 0.10 ^a^	0.38 ± 0.09 ^b^	0.39 ± 0.09 ^c^	<0.001
CRP (mg/dL)	0.22 ± 0.37 ^a^	0.23 ± 0.38 ^b^	0.24 ± 0.43 ^b^	0.001

HDL: high-density lipoprotein; LDL: low-density lipoprotein; FBG: fasting blood glucose; BUN: blood urea nitrogen; eGFR: estimated glomerular filtration rate; CRP: C-reactive protein. ^1^ Values are presented as mean ± SD for continuous variables or % for categorical variables. The *p*-value was analyzed by the chi-square test for categorical variables and one-way analysis of variance for continuous variables. Data with different superscript letters (^a, b, c^) indicate significantly different at *p* < 0.05 by Bonferroni post-hoc test.

**Table 4:** Characteristics of the subjects across tertiles of the veggie–fruit–grains dietary pattern (*n* = 56,476) ^1^.

	Veggie–Fruit–Grains Dietary Pattern			
	T1 (*n* = 18,825)	T2 (*n* = 18,826)	T3 (*n* = 18,825)	*p*-Value
Age (years)	38.1 ± 11.5 ^a^	41.2 ± 11.7 ^b^	44.5 ± 12.3 ^c^	<0.001
Sex (% female)	31.7	29.8	32.0	<0.001
Marital status (% married)	63.0	72.5	75.7	<0.001
Education (% high)	76.9	79.3	84.6	<0.001
Current smoker (% yes)	25.8	24.8	18.9	<0.001
Current drinker (% yes)	17.5	16.3	15.8	<0.001
Physical activity (% high)	30.1	32.8	35.6	<0.001
Body mass index (kg/m^2^)	25.5 ± 3.6 ^a^	25.1 ± 3.4 ^b^	25.0 ± 3.5 ^c^	0.005
Waist circumference (cm)	85.0 ± 10.5 ^a^	84.8 ± 10.9 ^b^	83.9 ± 13.1 ^b^	<0.001
Systolic blood pressure (mmHg)	124 ± 23 ^a^	122 ± 23 ^b^	121 ± 22 ^c^	<0.001
Diastolic blood pressure (mmHg)	78 ± 14 ^a^	77 ± 15 ^b^	76 ± 14 ^c^	<0.001
Total cholesterol (mmol/L)	5.20 ± 1.07 ^a^	5.19 ± 1.06 ^b^	5.13 ± 1.15 ^b^	<0.001
HDL cholesterol (mmol/L)	0.95 ± 0.68 ^a^	1.09 ± 0.63 ^b^	1.15 ± 0.60 ^c^	<0.001
LDL cholesterol (mmol/L)	3.16 ± 1.40 ^a^	3.03 ± 1.47 ^b^	2.70 ± 1.62 ^c^	<0.001
Triglycerides (mmol/L)	2.05 ± 1.09 ^a^	2.05 ± 1.22 ^a^	2.02 ± 1.36 ^b^	0.036
FBG (mmol/L)	5.76 ± 1.43 ^a^	5.66 ± 1.40 ^b^	5.45 ± 1.60 ^c^	<0.001
BUN (mmol/L)	5.17 ± 1.48 ^a^	4.97 ± 1.41 ^b^	4.60 ± 1.60 ^b^	<0.001
Creatinine (mol/L)	89.9 ± 31.2 ^a^	89.1 ± 27.6 ^b^	87.8 ± 29.6 ^c^	<0.001
eGFR (mL/min/1.73 m^2^)	84.6 ± 22.8 ^a^	84.9 ± 23.2 ^b^	86.0 ± 24.8 ^b^	<0.001
Uric acid (mmol/L)	0.39 ± 0.09 ^a^	0.38 ± 0.11 ^b^	0.36 ± 0.09 ^c^	<0.001
CRP (mg/dL)	0.24 ± 0.43 ^a^	0.22 ± 0.35 ^b^	0.21 ± 0.39 ^c^	<0.001

HDL: high-density lipoprotein; LDL: low-density lipoprotein; FBG: fasting blood glucose; BUN: blood urea nitrogen; eGFR: estimated glomerular filtration rate; CRP: C-reactive protein. ^1^ Values are presented as mean ± SD for continuous variables or % for categorical variables. The *p*-value was analyzed by chi-square test for categorical variables and one-way analysis of variance for continuous variables. Data with different superscript letters (^a, b, c^) indicate significantly different at *p* < 0.05 by Bonferroni post-hoc test.

**Table 5:** Characteristics of the subjects across tertiles of the meat–seafood–eggs dietary pattern (*n* = 56,476) ^1^.

	Meat–Seafood–Eggs Dietary Pattern			
	T1 (*n* = 18,825)	T2 (*n* = 18,826)	T3 (*n* = 18,825)	*p*-Value
Age (years)	43.7 ± 13.1 ^a^	41.4 ± 11.9 ^b^	38.6 ± 11.1 ^c^	<0.001
Sex (% female)	25.5	31.3	36.7	<0.001
Marital status (% married)	66.9	72.0	74.1	<0.001
Education (% high)	87.1	84.7	68.5	<0.001
Current smoker (% yes)	15.7	23.6	30.2	<0.001
Current drinker (%yes)	11.0	16.9	21.7	<0.001
Physical activity (% high)	34.7	32.5	31.3	<0.001
Body mass index (kg/m^2^)	24.8 ± 3.6 ^a^	25.0 ± 3.5 ^b^	25.7 ± 3.5 ^c^	0.003
Waist circumference (cm)	83.3 ± 14.6 ^a^	84.5 ± 14.6 ^b^	85.9 ± 11.1 ^c^	<0.001
Systolic blood pressure (mmHg)	122 ± 22 ^a^	122 ± 22 ^a^	123 ± 23 ^b^	<0.001
Diastolic blood pressure (mmHg)	77 ± 15 ^a^	77 ± 14 ^a^	77 ± 14 ^a^	0.002
Total cholesterol (mmol/L)	5.14 ± 1.16 ^a^	5.18 ± 1.07 ^a^	5.20 ± 1.07 ^b^	<0.001
HDL cholesterol (mmol/L)	1.08 ± 0.63 ^a^	1.06 ± 0.63 ^b^	1.05 ± 0.66 ^c^	<0.001
LDL cholesterol (mmol/L)	2.90 ± 1.54 ^a^	2.95 ± 1.50 ^b^	3.04 ± 1.47 ^c^	<0.001
Triglycerides (mmol/L)	2.00 ± 1.12 ^a^	2.02 ± 1.15 ^b^	2.10 ± 1.39 ^c^	<0.001
FBG (mmol/L)	5.55 ± 1.69 ^a^	5.63 ± 1.35 ^b^	5.69 ± 1.39 ^c^	<0.001
BUN (mmol/L)	4.77 ± 1.70 ^a^	4.95 ± 1.39 ^b^	5.02 ± 1.39 ^c^	<0.001
Creatinine (mol/L)	87.2 ± 30.5 ^a^	89.2 ± 26.8 ^b^	90.3 ± 31.0 ^c^	<0.001
eGFR (mL/min/1.73 m^2^)	85.5 ± 22.8 ^a^	85.3 ± 23.0 ^a^	84.8 ± 23.6 ^b^	0.001
Uric acid (mmol/L)	0.36 ± 0.10 ^a^	0.38 ± 0.09 ^b^	0.39 ± 0.09 ^c^	<0.001
CRP (mg/dL)	0.22 ± 0.38 ^a^	0.22 ± 0.38 ^b^	0.25 ± 0.43 ^c^	<0.001

HDL: high-density lipoprotein; LDL: low-density lipoprotein; FBG: fasting blood glucose; BUN: blood urea nitrogen; eGFR: estimated glomerular filtration rate; CRP: C-reactive protein. ^1^ Values are presented as mean ± SD for continuous variables or % for categorical variables. The *p*-value was analyzed by chi-square test for categorical variables and one-way analysis of variance for continuous variables. Data with different superscript letters (^a, b, c^) indicate significantly different at *p* < 0.05 by Bonferroni post-hoc test.

**Table 6:** Characteristics of the subjects across tertiles of the milk–dairy dietary pattern (*n* = 56,476) ^1^.

	Milk–Dairy Dietary Pattern			
	T1 (*n* = 18,825)	T2 (*n* = 18,826)	T3 (*n* = 18,825)	*p*-Value
Age (years)	40.7 ± 12.7 ^a^	40.9 ± 11.7 ^b^	42.2 ± 12.2 ^c^	<0.001
Sex (% female)	29.0	30.7	33.7	<0.001
Marital status (% married)	68.7	70.7	72.7	<0.001
Education (% high)	70.2	85.1	85.2	<0.001
Current smoker (% yes)	18.9	24.1	26.5	<0.001
Current drinker (% yes)	14.7	16.4	18.5	<0.001
Physical activity (% high)	30.1	33.2	35.2	<0.001
Body mass index (kg/m^2^)	25.5 ± 3.9 ^a^	25.1 ± 3.7 ^b^	25.1 ± 3.8 ^c^	0.001
Waist circumference (cm)	84.9 ± 13.1 ^a^	84.8 ± 10.6 ^a^	84.0 ± 10.9 ^b^	<0.001
Systolic blood pressure (mmHg)	123 ± 23 ^a^	122 ± 22 ^b^	122 ± 22 ^b^	<0.001
Diastolic blood pressure (mmHg)	78 ± 15 ^a^	77 ± 14 ^b^	77 ± 15 ^b^	<0.001
Total cholesterol (mmol/L)	5.19 ± 1.06 ^a^	5.18 ± 1.07 ^b^	5.15 ± 1.17 ^c^	<0.001
HDL cholesterol (mmol/L)	1.04 ± 0.62 ^a^	1.06 ± 0.64 ^b^	1.11 ± 0.60 ^c^	<0.001
LDL cholesterol (mmol/L)	3.07 ± 1.46 ^a^	3.03 ± 1.46 ^b^	2.79 ± 1.57 ^c^	<0.001
Triglycerides (mmol/L)	2.13 ± 1.41 ^a^	2.03 ± 1.16 ^b^	1.96 ± 0.99 ^c^	0.027
FBG (mmol/L)	5.67 ± 1.34 ^a^	5.63 ± 1.33 ^b^	5.55 ± 1.74 ^b^	<0.001
BUN (mmol/L)	4.97 ± 1.38 ^a^	4.93 ± 1.41 ^b^	4.84 ± 1.71 ^c^	<0.001
Creatinine (mol/L)	90.0 ± 27.0 ^a^	88.8 ± 24.4 ^b^	88.0 ± 36.4 ^c^	<0.001
eGFR (mL/min/1.73 m^2^)	84.5 ± 24.3 ^a^	85.5 ± 23.5 ^b^	85.7 ± 23.1 ^b^	<0.001
Uric acid (mmol/L)	0.38 ± 0.11 ^a^	0.38 ± 0.09 ^a^	0.37 ± 0.09 ^b^	<0.001
CRP (mg/dL)	0.25 ± 0.45 ^a^	0.22 ± 0.36 ^b^	0.22 ± 0.38 ^c^	<0.001

HDL: high-density lipoprotein; LDL: low-density lipoprotein; FBG: fasting blood glucose; BUN: blood urea nitrogen; eGFR: estimated glomerular filtration rate; CRP: C-reactive protein. ^1^ Values are presented as mean ± SD for continuous variables or % for categorical variables. The *p*-value was analyzed by chi-square test for categorical variables and one-way analysis of variance for continuous variables. Data with different superscript letters (^a, b, c^) indicate significantly different at *p* < 0.05 by Bonferroni post-hoc test.

**Table 7:** Multiple linear regression analysis of the association between dietary patterns and kidney function parameters ^1^.

BUN (mmol/L)	T1 (Ref)	T2	T3	*p*-Value
Processed food–sweets dietary pattern				
Model 1 ^a^	4.83 (4.81–5.02)	4.88 (4.86–4.90)	4.99 (4.97–5.01)	<0.001
Model 2 ^b^	4.84 (4.82–5.03)	4.88 (4.86–4.90)	4.98 (4.96–5.01)	<0.001
Veggie–fruit–grains dietary pattern				
Model 1 ^a^	4.96 (4.94–4.98)	4.96 (4.94–4.98)	4.78 (4.76–4.80)	<0.001
Model 2 ^b^	4.96 (4.94–4.98)	4.96 (4.94–4.98)	4.78 (4.76–4.80)	<0.001
Meat–seafood–eggs dietary pattern				
Model 1 ^a^	4.76 (4.74–4.79)	4.92 (4.90–4.94)	5.03 (5.02–5.06)	<0.001
Model 2 ^b^	4.77 (4.75–4.79)	4.92 (4.90–4.94)	5.04 (5.02–5.06)	<0.001
Milk–dairy dietary pattern				
Model 1 ^a^	5.05 (5.02–5.07)	4.83 (4.82–4.86)	4.82 (4.80–4.84)	<0.001
Model 2 ^b^	5.05 (5.02–5.07)	4.84 (4.82–4.86)	4.82 (4.80–4.84)	<0.001
**Creatinine (mol/L)**				
Processed food–sweets dietary pattern				
Model 1 ^a^	86.6 (86.1–87.1)	86.9 (86.5–87.3)	87.7 (87.3–88.2)	0.005
Model 2 ^b^	86.6 (86.2–87.1)	86.9 (86.5–87.3)	87.7 (87.2–88.1)	0.008
Veggie–fruit–grains dietary pattern				
Model 1 ^a^	89.2 (88.7–89.6)	88.5 (88.1–89.0)	87.5 (87.1–87.9)	<0.001
Model 2 ^b^	89.2 (88.7–89.6)	88.4 (87.9–88.9)	87.5 (87.1–88.0)	<0.001
Meat–seafood–eggs dietary pattern				
Model 1 ^a^	87.6 (87.1–88.0)	88.1 (87.7–88.5)	88.9 (88.4–89.3)	<0.001
Model 2 ^b^	87.4 (87.3–88.3)	88.1 (87.7–88.5)	88.9 (88.4–89.3)	<0.001
Milk–dairy dietary pattern				
Model 1 ^a^	88.8 (88.4–89.3)	88.1 (87.7–88.5)	87.7 (87.2–88.1)	0.001
Model 2 ^b^	88.7 (88.2–89.2)	88.1 (87.7–88.5)	87.8 (87.4–88.2)	0.004
**eGFR (mL/min/1.73 m^2^)**				
Processed food–sweets dietary pattern				
Model 1 ^a^	85.7 (85.4–86.1)	85.0 (84.7–85.2)	84.8 (84.6–85.1)	0.007
Model 2 ^b^	85.6 (85.4–85.9)	85.0 (84.7–85.4)	84.8 (84.6–85.1)	0.006
Veggie–fruit–grains dietary pattern				
Model 1 ^a^	84.6 (84.4–84.9)	85.0 (84.7–85.3)	85.8 (85.5–86.0)	0.003
Model 2 ^b^	84.6 (84.4–84.9)	85.0 (84.7–85.2)	85.8 (85.6–86.1)	0.004
Meat–seafood–eggs dietary pattern				
Model 1 ^a^	85.9 (85.7–86.1)	84.9 (84.7–85.1)	84.6 (84.4–84.9)	<0.001
Model 2 ^b^	85.8 (85.6–86.1)	85.0 (84.7–85.2)	84.7 (84.4–85.0)	<0.001
Milk–dairy dietary pattern				
Model 1 ^a^	84.5 (84.3–84.7)	85.1 (84.8–85.3)	86.0 (85.7–86.1)	<0.001
Model 2 ^b^	84.6 (84.4–84.9)	85.1 (84.8–85.3)	85.9 (85.6–86.2)	<0.001
**Uric acid (mmol/L)**				
Processed food–sweets dietary pattern				
Model 1 ^a^	0.37 (0.37–0.38)	0.38 (0.38–0.38)	0.39 (0.39–0.39)	<0.001
Model 2 ^b^	0.37 (0.37–0.38)	0.38 (0.38–0.38)	0.39 (0.39–0.39)	<0.001
Veggie–fruit–grains dietary pattern				
Model 1 ^a^	0.39 (0.39–0.39)	0.38 (0.38–0.38)	0.36 (0.36–0.36)	<0.001
Model 2 ^b^	0.39 (0.39–0.39)	0.38 (0.38–0.38)	0.37 (0.37–0.37)	<0.001
Meat–seafood–eggs dietary pattern				
Model 1 ^a^	0.36 (0.36–0.37)	0.37 (0.37–0.37)	0.38 (0.38–0.39)	<0.001
Model 2 ^b^	0.37 (0.36–0.37)	0.37 (0.37–0.37)	0.39 (0.39–0.39)	<0.001
Milk–dairy dietary pattern				
Model 1 ^a^	0.38 (0.37–0.38)	0.38 (0.38–0.38)	0.37 (0.37–0.38)	<0.001
Model 2 ^b^	0.38 (0.37–0.38)	0.38 (0.38–0.38)	0.37 (0.37–0.37)	<0.001

BUN: blood urea nitrogen; eGFR: estimated glomerular filtration rate. ^1^ Values are expressed as mean (95% CI). ^a^ Model 1 was adjusted for age, sex, marital status, education, current smoking, current drinking, and physical activity. ^b^ Model 2 was adjusted for age, sex, marital status, education, current smoking, current drinking, physical activity, and the components of metabolic syndrome.

## Data Availability

The data that support the findings of this study are available from Mei Jau (MJ) Health Institute, but restricted for research use only. The data are not publicly available. Data are available from the authors upon reasonable request and with permission of MJ Health Institute.

## Supplementary Materials

The following is available online at https://www.mdpi.com/2072-6643/13/1/40/s1, Table S1: Food items and description of servings in each food group used to derive dietary patterns.
