# Supplementary material for: Association between Dietary Patterns and Kidney Function Parameters in Adults with Metabolic Syndrome: A Cross-Sectional Study
Source: Nutrients. 2020 Dec 24;13(1):40. doi: 10.3390/nu13010040 (PMC7823384; doi:10.3390/nu13010040)
Supplement: Supplementary file 1 [file nutrients-13-00040-s001.pdf]

Supplementary Material: Association between Dietary Patterns and Kidney Function Parameters in Adults with Metabolic Syndrome: A Cross-Sectional Study

Ahmad Syauqy [1,2], Chien-Yeh Hsu [3,4], Hsiu-An Lee [5], Hsiao-Hsien Rau [6] and Jane C.-J. Chao [1,4,7,*]

Table S1. Food items and description of servings in each food group used to derive dietary patterns.

Food Groups	Food Items and Servings
Milk	Fresh milk, yogurt, powdered milk
	(1 glass is equivalent to 240 mL of fresh milk, 240 mL of drying yogurt, or 4 tablespoons of powdered milk)
Dairy products	Yogurt, cheese
	(1 serving is equivalent to 1 slice of yogurt or cheese)
Eggs	Chicken egg, duck egg, quail eggs
	(1 serving is equivalent to 1 chicken or duck egg, or 5 quail eggs)
Meat	Pork, chicken, duck, beef, veal, lamb
	(1 serving is equivalent to 1 pork or beef steak, approximately palm size, or 1 chicken leg, 1 hamburger patty, or 4 tablespoons of other lean meat)
Seafood	Fish, shrimps, oysters
	(1 serving is equivalent to 2 ounces of fish, 4 slices of row fish, 4 shrimps, or 16 oysters)
Organ meats	Liver, kidneys, heart, intestines
	(1 serving is equivalent to a half bowl of pork or chicken liver, 8 chicken hearts, or 4 tablespoon of cooked pork intestines)
Legumes/soy products	Tofu, soybean milk, dried beancurd
	(1 serving is equivalent to a half box of packaged tofu, 240 mL of soybean milk, or 2 pieces of dried beancurd)
Light-colored vegetables	Cabbage, pechay, cucumber, radish
	(1 serving is equivalent to a bowl of vegetables)
Dark-colored vegetables	Carrots, spinach, squash, tomatoes
	(1 serving is equivalent to a bowl of vegetables)
Vegetables in oil/dressing	Vegetables are fried with oil or with salad dressing
	(This is mainly for calculating the fat intake and total calories)
Fruit	Apple, grapefruit, guava, kiwi, papaya, watermelon, lychees, grapes, dragon eyes
	(1 serving is equivalent to a half medium sized apple, grapefruit, or guava, 1 orange or kiwi, a half kilo of papaya or watermelon, 5 lychees, or 12 grapes or dragon eyes)

Rice/flour products	Rice, noodles, bread, twisted cruller<br>(1 serving is equivalent to a bowl of rice, two bowls of noodles, four slices of thin bread, 1 round bread, or 2 sets of baked wheat bread or twisted cruller)
Whole grains	Whole wheat bread, brown rice, mixed grains, oatmeal<br>(1 serving is equivalent to 4 slices of whole wheat bread, a bowl of brown rice or mixed grains, or 8 tablespoons of oatmeal)
Fried rice/flour products	Fried rice, fried noodles, fried rice noodles, baked wheat bread oil?<br>(1 serving is equivalent to a bowl of fried rice, fried noodles, or fried rice noodles, or 2 sets of baked wheat bread or twisted cruller)
Root crops	Sweet potato, potato, taro, corn<br>(1 serving is equivalent to a bowl of root crops)
Refined dessert	Red bean bread, butter bread, cake, cookies<br>(1 serving equivalent to 1 red bean bread or butter bread, 1 slice of cake, or 15 cookies)
Jam/honey	Jam, honey<br>(1 serving is equivalent to 2 teaspoon of jam or honey)
Sugary drinks	Coffee, tea, fruit juice, soft drinks, green bean soup<br>(1 serving is equivalent to 240 mL or 1 glass)
Deep-fried food	Deep-fried potatoes, seafood, chicken, duck<br>(1 serving is equivalent to a half bowl)
Processed food	Ham, sausage, canned food, preserved fish<br>(1 serving is equivalent to 1 tablespoon preserved vegetables, a half ounce of sausage, 2 slices of ham, or 1 tablespoon of canned meat)
Instant noodles	Instant noodles, instant vermicelli, instant rice<br>(1 serving is equivalent to 1 bowl or a pack of instant noodles)
Sauce	Soy sauce, ketchup, hot sauce, vinegar, pepper salt<br>(1 serving is equivalent to 2 teaspoons of soy sauce, ketchup, hot sauce, or vinegar, or 1 teaspoon of pepper salt)
